# Xanthine Oxidase Inhibitory Activity and Chemical Composition of *Pistacia chinensis* Leaf Essential Oil

**DOI:** 10.3390/pharmaceutics14101982

**Published:** 2022-09-20

**Authors:** Chi-Ya Huang, Yu-Yi Chang, Shang-Tzen Chang, Hui-Ting Chang

**Affiliations:** 1 Agricultural Technology Research Institute, Hsinchu 300110, Taiwan; 2 School of Forestry and Resource Conservation, National Taiwan University, Taipei 10617, Taiwan

**Keywords:** essential oil, gout, *Pistacia chinensis*, terpenoids, xanthine oxidase inhibitory activity

## Abstract

Gout is a common metabolic disease caused by abnormal purine metabolism that promotes the formation and deposition of monosodium urate crystals within joints that causes acute arthritis and can seriously affect the daily life of patients. *Pistacia chinensis* is one of the traditional medicinal plants of the Anacardiaceae family, and there have been many studies on its biological activity, including anti-inflammatory, antidepressant, antibacterial, antioxidant, and hypoglycemic activities. The aim of this study was to evaluate the antigout effect of *P. chinensis* leaf essential oil and its constituents through xanthine oxidase inhibition. Leaf essential oil showed good xanthine oxidase inhibitory activity for both substrates, hypoxanthine and xanthine. Six fractions were obtained from open column chromatography, and fraction E1 exhibited the best activity. The constituents of leaf essential oil and fraction E1 were analyzed by GC-MS. The main constituents of both leaf essential oil and fraction E1 were limonene and 3-carene; limonene showed a higher inhibitory effect on xanthine oxidase. Based on the enzyme kinetic investigation, limonene was the mixed-type inhibitor against xanthine oxidase. The results revealed that *Pistacia chinensis* leaf essential oil and limonene have the potential to act as natural remedies for the treatment of gout.

## 1. Introduction

Gout is a disorder of purine metabolism resulting from the high uric acid level in serum (hyperuricaemia), which causes urate or uric acid crystal deposition within the joints. The symptoms of acute gout include severe pain, swelling, and redness in the joints, and the disease can seriously affect the daily life and diet of patients. Purine catabolic enzymes convert dietary and endogenous purines to hypoxanthine and xanthine by complex enzyme systems. Xanthine oxidase is a key enzyme that catalyzes the oxidation of hypoxanthine to xanthine, and further catalyzes xanthine to uric acid [1,2,3,4]. Allopurinol, a xanthine oxidase inhibitor, has been used in treatments for hyperuricemia and gout; febuxostat, topiroxostat, uricosurics, probenecid, etc., are relatively new drugs. Some therapeutic agents may cause adverse effects; for example, febuxostat can cause side effects such as diarrhea, nausea, and elevation of liver enzymes [5,6,7].

Researchers continue to explore xanthine oxidase inhibitors from plant natural products [8,9,10,11,12,13]. *Cinnamomum cassia* twig extract was found to exhibit the xanthine oxidase inhibitory activity [14]. The extracts of *Artemisia vulgaris*, *Caesalpinia sappan*, *Blumea balsamifera*, *Chrysanthemum sinense*, and *Tetracera scandens* showed inhibition effects against xanthine oxidase [15]. Muraoka and Miura stated that phytic acid, abundant in plants, nuts, cereals, etc., can inhibit the formation of uric acid, with an IC_50_ value of about 30 mM, using xanthine as the substrate [16].

The genus *Pistacia* (Anacardiaceae) is widely distributed in North and Central America, Africa, southern Europe, and Asia. *P. vera* is the species in this genus famous for the production of edible pistachio nuts [17,18]. The bioactivities of folk medicinal plants in this genus include wound healing, antioxidant, anti-inflammatory, antimicrobial, hypoglycemic, neuroprotective, antidiarrheal, analgesic, antipyretic, hypoglycemic, hypotensive, cardioprotective, anticancer, antileishmanial effects, etc. [17,19,20,21,22,23,24,25,26]. Assimopoulou and Papageorgiou reported that 36 triterpenoids were identified from *P. lentiscus* var. chia resin by GC-MS analysis; the major compounds were isomasticadienonic acid, masticadienonic acid, and 28-norolean-17-en-3-one [27].

Antimicrobial, including antibacterial and antifungal, activities were found in the essential oils or extracts from *P. lentiscus* leaf, berry and bark, *P. atlantica* mastic gum, and *P. atlantica* leaf and fruit [20,21,23,28,29,30]. Antioxidant activity has been reported in *P. atlantica* subsp. *kurdica* fruit extract, *P. atlantica* var. *mutica* kernel oil, pistachio kernel extract and *P. lentiscus* leaf and fruit extracts [24,31,32,33]. Anticancer activities have been found in *P. lentiscus* leaf extract, and essential oil of mastic gums of *P. lentiscus* var. *chia* and *P. lentiscus* bark [34,35].

*P. chinensis* is one of the common *Pistacia* species distributed throughout Asia. The traditional uses of *P. chinensis* include ornamental purposes, wood, seed oil, and folk remedies for diarrhea, sore throat, detoxification, etc. De Pooter et al. reported the main constituents of the chemical compositions of *P. chinensis* leaf essential oils grown in Egypt were *trans*-8-ocimene, limonene, etc. [36]. Two 3-3″-dimeric 4-phenyldihydrocoumarin compounds were isolated from the twig extract of *P. chinensis* and proven to possess estrogen-like activity [37].

In this study, the xanthine oxidase inhibitory activity of *P. chinensis* leaf essential oil and its fractions were evaluated. The chemical composition of leaf essential oil and its active fraction were analyzed by gas chromatography-mass spectrometry. Enzyme kinetic study was used to elucidate the inhibition type of active constituent on xanthine oxidase.

## 2. Materials and Methods

### 2.1. Hydrodistillation of Leaf Essential Oil

Leaves of *Pistacia chinensis*, around 35 years old, were harvested from National Taiwan University (NTU), Taipei, Taiwan. The specimen of plant material (PC1014) was deposited in the laboratory of Chemical Utilization of Biomaterials, School of Forestry and Resource Conservation, NTU. Essential oil from fresh leaves was obtained by hydrodistillation using a Clevenger apparatus for 8 h [38,39,40]. Leaf essential oil was placed in a dark glass bottle and stored in a refrigerator at 4 °C.

### 2.2. Column Chromatography and Thin Layer Chromatography

*P. chinensis* leaf essential oil (50 g) was further subjected to classical preparative silica gel column chromatography (CC) with a gradient elution of *n*-hexane and ethyl acetate of increasing polarity, and then separated into six fractions (E1–E6) using thin-layer chromatography (TLC) to analyze the elution profiles at both 254 nm and 365 nm UV light [41,42,43].

### 2.3. GC-MS Analysis of Leaf Essential Oil and Fraction E1

Chemical constituents in leaf essential oil and fraction E1 were analyzed using a Thermo Trace GC Ultra gas chromatograph equipped with a Polaris Q MSD mass spectrometer (Thermo Fisher Scientific, Austin, TX, USA). Analyte (1 μL) was injected into the capillary column (DB-5MS, Crossbond 5% phenyl methyl polysiloxane, 30 m length × 0.25 mm i.d. × 0.25 µm film thickness). The GC column temperature program was set as follows: initial temperature of 60 °C for 3 min; 2 °C/min up to 120 °C with a 3 min hold; 3 °C/min up to 180 °C; 10 °C/min up to 250 °C with a 5 min hold. The flow rate of the carrier gas, helium, was 1 mL/min and the split ratio was 1:10. The compound was characterized by comparing the mass spectra (*m*/*z* 50–650 amu) with library databases, including National Institute of Standards and Technology (NIST) and Wiley, and Arithmetic index (AI) [44]. The quantification of constituents was analyzed by integrating the peak area of the chromatogram [45,46].

### 2.4. Xanthine Oxidase Assay

Xanthine oxidase inhibitory activity of specimens was evaluated by using in vitro spectrophotometric analysis [11,47]. Both hypoxanthine and xanthine were used as the substrate in the assay, respectively. We added 117 µL of potassium phosphate buffer (50 mM, pH 7.8), 3 µL of specimen solution, and 60 µL of 0.025 unit/mL xanthine oxidase (EC 1.1.3.22) and mixed well in a 96-well microplate, and the mixture was incubated for 10 min at room temperature (25 °C). We added 100 µL of 0.15 mM substrate (hypoxanthine/xanthine) into the well, and the solution was incubated in the dark for 30 min at 37 °C. Then, 20 µL of 1 N HCl was added to stop the reaction. Absorbance at 290 nm of each well was measured using an ELISA (enzyme-linked immunosorbent assay) microplate reader (SPECTROstar Nano, BMG LABTECH, Offenburg, Germany) after incubation. Allopurinol, a therapeutic agent for gout, was used as the positive control. All the experiments were performed in triplicate. Inhibition of the xanthine oxidase inhibitory activity was determined by the following formula: Inhibition (%) = [(A_control_ − A_control’s blank_) − (A_sample_ − A_sample’s blank_)/(A_control_ − A_control’s blank_)] × 100. The IC_50_, half maximal inhibitory concentration, was calculated from the concentration-response curve of the specimen.

### 2.5. Enzyme Kinetic Study

The Lineweaver–Burk reciprocal plot of the reaction rate and concentration of the substrate was used to determine kinetic parameters for enzyme kinetic study, and to evaluate the interaction of the compound on the affinity of the substrate and enzyme. The concentration of xanthine oxidase was constantly kept at 0.025 unit/mL, and the concentration of substrate (hypoxanthine/xanthine) was varied at the range of 0.0125–0.20 mM. The reaction was similar to the xanthine oxidase assay as described above; 117 µL of potassium phosphate buffer (50 mM, pH 7.8), 3 µL of specimen solution, 60 µL of 0.025 unit/mL xanthine oxidase, and 100 µL of the substrate (hypoxanthine/xanthine) was added into the 96-well microplate and mixed well, and kinetic measurements of the solution were immediately taken for a period of 3 min at 290 nm at 37 °C. Kinetic parameters, including Michaelis–Menten constant (K_m_) and maximum velocity (V_max_), were measured from the Lineweaver–Burk linear equation [48,49]. Main types of inhibition of enzyme inhibitors included competitive inhibition, noncompetitive inhibition, uncompetitive inhibition, and mixed inhibition.

### 2.6. Statistical Analysis

Statistical analysis of the data was performed by SPSS (Statistical Product and Service Solutions) (Chicago, IL, USA) Version 16 using Scheffe’s multiple comparison test (a post hoc multiple comparison method). The confidence interval was computed at the confidence level of 95%.

## 3. Results and Discussion

### 3.1. Xanthine Oxidase Inhibition Effects of P. chinensis Leaf Essential Oil and Its Fractions

Leaf essential oil of *P. chinensis* was obtained by hydrodistillation, and the yield of leaf essential oil was 0.53 ± 0.06% (on dry matter basis, *n* = 3). Table 1 shows the inhibitory effects of *P. chinensis* leaf essential oil and the positive control, allopurinol, against xanthine oxidase. The IC_50_ values of leaf essential oil were 43.52 and 55.40 μg/mL when using hypoxanthine and xanthine as the substrate, respectively. For allopurinol, the IC_50_ values were 0.13 and 0.11 μg/mL with hypoxanthine and xanthine as the substrate, respectively. Ahmad et al. reported the xanthine oxidase inhibitory activities of aqueous extract and ethanolic extract from *P. integerrima* leaf; the IC_50_ values of the aqueous and ethanolic extracts were 85 and 60 μg/mL, respectively, when using hypoxanthine as the substrate [47]. Leaf and bark extracts of *Erythrina variegata* were demonstrated to exhibit inhibitory activity of xanthine oxidase with IC_50_ values of 84.75 and 52.75 μg/mL, respectively, using xanthine as the substrate [50]. Our results revealed that *P. chinensis* leaf essential oil exhibited potent xanthine oxidase inhibitory activity.

**Figure 1:**
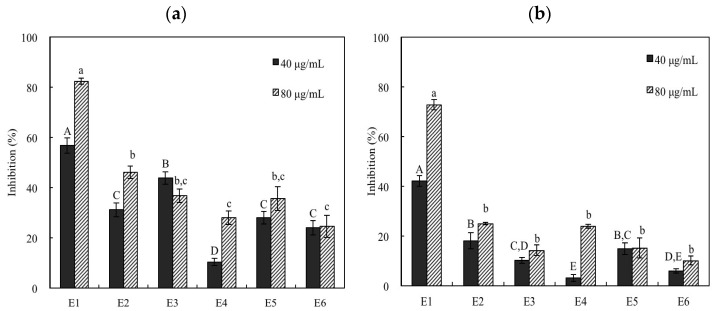
Xanthine oxidase inhibition effects of six fractions of leaf essential oil. (**a**) Hypoxanthine as the substrate; (**b**) xanthine as the substrate. Results are mean ± SD (*n* = 3). Different letters in the figure indicate significantly different inhibition percentages between specimens at the same concentration at the level of *p* < 0.05 according to Scheffe’s test.

The leaf essential oil was further fractionated into six fractions (E1 to E6) by silica gel column chromatography. The yields of each fraction were 57.93% (E1, elution with 100% *n*-hexane), 27.50% (E2, elution with 5% ethyl acetate/95% *n*-hexane), 2.45% (E3, elution with 20% ethyl acetate/80% *n*-hexane), 7.39% (E4, elution with 50% ethyl acetate/50% *n*-hexane), 3.22% (E5, elution with 100% ethyl acetate), and 1.51% (E6, elution with 100% ethyl acetate). Among these subfractions, fraction E1 exhibited higher xanthine oxidase inhibitory effect in both substrate, hypoxanthine and xanthine, assays (Figure 1). There were statistically significant differences (*p* < 0.05) between fraction E1 and the other fractions. The IC_50_ values of fraction E1 were 40.55 and 51.84 μg/mL when using hypoxanthine and xanthine as the substrate, respectively (Table 2). The IC_50_ values of the other fractions (E2–E6) were higher than 80 μg/mL.

### 3.2. Chemical Composition Analysis of P. chinensis Leaf Essential Oil and Fraction E1

**Figure 2:**
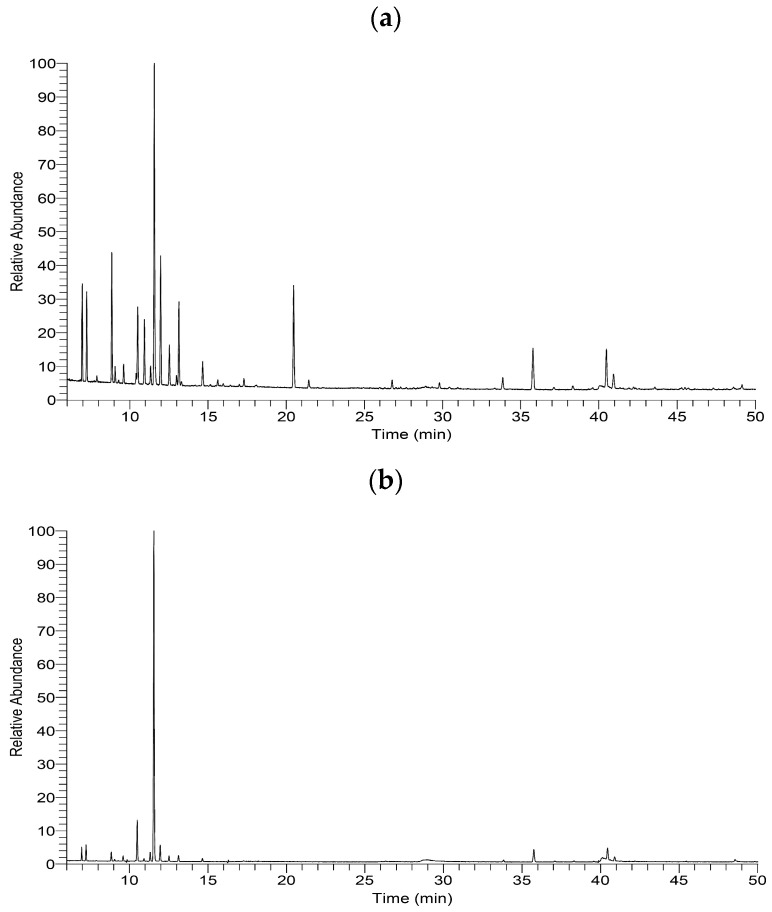
Gas chromatograms of *Pistacia chinensis* leaf essential oil and fraction E1. (**a**) Leaf essential oil; (**b**) fraction E1.

Figure 2a shows the gas chromatogram of *P. chinensis* leaf essential oil. The major constituents of *P. chinensis* leaf essential oil were limonene (24.01%), terpinen-4-ol (9.18%), *cis*-β-ocimene (9.15%), sabinene (8.25%), γ-terpinene (6.30%), 3-carene (5.32%), α-pinene (5.18%), and β-caryophyllene (5.08%) (Table 3). The chemical constituents of *P. chinensis* leaf essential oil were classified into monoterpenes (71.44%), oxygenated monoterpenes (9.89%), and sesquiterpenes (6.65%); the main C-skeletons included menthane, myrcane, thujane, carane, pinane, and caryophyllane skeletons.

De Pooter et al. analyzed the chemical compositions of leaf essential oils from three *Pistacia* species (*P. khinjuk*, *P. chinensis* and *P. lentiscus*) grown in Egypt [36]. They also found that limonene was rich in the leaf essential oil of *P. chinensis* grown in Egypt; the content of limonene was 26.5%. There were also some differences in the compositions of leaf essential oils in different growing regions.

The major constituents of active fraction E1 (Figure 2b) were limonene (61.06%) and 3-carene (7.04%), as listed in Table 3; the minor constituents were β-caryophyllene (3.72%), *cis*-β-ocimene (2.91%), α-pinene (2.38%), *p*-cymene (1.63%), sabinene (1.50%), β-myrcene (1.19%), and γ-terpinene (1.07%). All constituents of fraction E1 belonged to the monoterpenes (80.26%) and sesquiterpenes (3.72%).

### 3.3. Xanthine Oxidase Inhibition Activity and Enzyme Kinetic Study of Main Constituents of Fraction E1

The xanthine oxidase inhibitory activity of main constituents in the active fraction E1 is represented in Table 4. The IC_50_ values of limonene and 3-carene were 37.69 μg/mL (0.28 mM) and 110.34 μg/mL (0.81 mM), respectively, using hypoxanthine as the substrate. When xanthine was the substrate, limonene was still effective, with an IC_50_ value of 48.04 μg/mL (0.35 mM); 3-carene did not display activity against xanthine oxidase. Limonene showed inhibitory effects of reducing the formation of uric acid in both substrates.

Two flavonoids, apigenin and rutin, were reported to exhibit xanthine oxidase inhibition activity with IC_50_ values of 35 and 61 μg/mL, respectively, using hypoxanthine as the substrate [47]. Priyatno et al. analyzed the xanthine oxidase inhibition activity of ethyl acetate extract from snake fruit (*Salacca edulis*), and found the active compound, 2-metyl ester-1H-pyrrole-4-carboxilyc acid, showed the best efficacy with an IC_50_ value of 48.86 μg/mL, using xanthine as the substrate [51].

**Figure 3:**
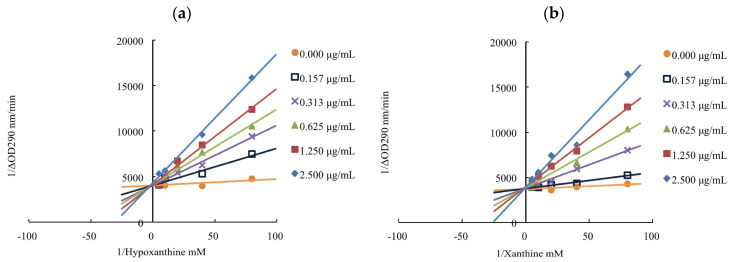
Lineweaver–Burk plots of allopurinol: (**a**) hypoxanthine as the substrate; (**b**) xanthine as the substrate.

The main modes of enzyme inhibition include competitive, uncompetitive, noncompetitive, and mixed types [16,52]. The inhibition mechanisms of allopurinol and limonene were elucidated by an enzyme kinetic study. Figure 3 shows the Lineweaver–Burk plots of the positive control, allopurinol, with both substrates, hypoxanthine and xanthine. The linear regression lines of allopurinol under different dosage levels had the same intercept on the y-axis and increasing slopes. Table 5 shows the kinetic parameters of allopurinol against xanthine oxidase; an increase in K_m_ and a constant in V_max_ were observed. The results indicated the inhibition type of allopurinol on xanthine oxidase was a competitive model to suppress the production of uric acid. Chen et al. reported that allopurinol exhibited competitive-type inhibition, which is consistent with our results [52]. Competitive inhibition demonstrated that allopurinol would bind to free xanthine oxidase with strong affinity, and prevent substrate, hypoxanthine/xanthine, binding to xanthine oxidase.

**Figure 4:**
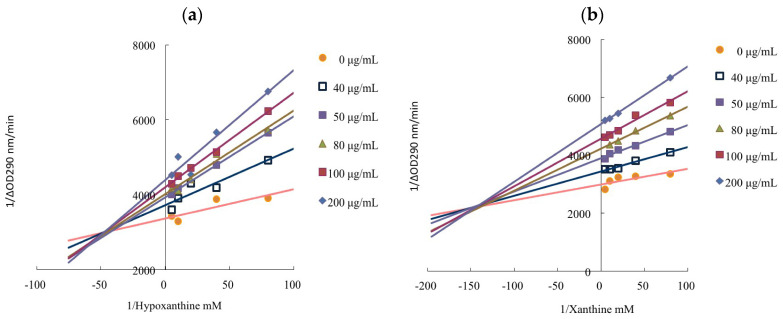
Lineweaver–Burk plots of limonene: (**a**) hypoxanthine as the substrate; (**b**) xanthine as the substrate.

The Lineweaver–Burk plots and kinetic parameters of limonene are shown in Figure 4 and Table 6. In the presence of limonene, an increase in Km and a decrease in V_max_ were observed. It revealed limonene was a mixed-type inhibitor to reduce the formation of uric acid. The inhibition type of quercetin, a versatile flavonoid, against that of xanthine oxidase was also a mixed type, containing competitive and noncompetitive types, as demonstrated in previous studies [48,49].

## 4. Conclusions

Gout is a metabolic disease caused by abnormal purine metabolism, which promotes the formation of uric acid. A daily diet with purine-rich foods might easily cause gout, and it is imperative to develop medicines or remedies with low side effects for gout therapy. The xanthine oxidase inhibitory effects of *P. chinensis* leaf essential oil and its constituents were evaluated in this study. The IC_50_ values of leaf essential oil against xanthine oxidase were 43.52 and 55.40 μg/mL when using hypoxanthine and xanthine as the substrate, respectively. Among the examined fractions separated from leaf essential oil, fraction E1 had the best xanthine oxidase inhibition activity to reduce the formation of uric acid with IC_50_ values of 40.55 and 51.84 μg/mL when using hypoxanthine and xanthine as the substrate, respectively. Limonene, a major constituent of fraction E1, showed an inhibitory effect on xanthine oxidase with IC_50_ values of 37.69 and 48.04 μg/mL using hypoxanthine and xanthine as the substrate, respectively. Through an enzyme kinetic study, we found that limonene was a mixed type inhibitor of xanthine oxidase for both substrates. The results indicated *P. chinensis* leaf essential oil and limonene have potential as natural xanthine oxidase inhibitors for the treatment of gout. Further investigation is required for potential clinical application.

## Figures and Tables

**Table 1:** IC_50_ values of *Pistacia chinensis* leaf essential oil against xanthine oxidase.

Specimen	IC_50_ (μg/mL)	
	Hypoxanthine as the Substrate	Xanthine as the Substrate
Leaf essential oil	43.52 ± 2.14 ^a^	55.40 ± 3.49 ^A^
Allopurinol *	0.13 ± 0.01 ^b^	0.11 ± 0.01 ^B^

Results are mean ± SD (*n* = 3). * Positive control. Different letters in the table indicate significantly different IC_50_ values between specimens in the same substrate at the level of *p* < 0.05 according to Scheffe’s test.

**Table 2:** IC_50_ values of six fractions against xanthine oxidase.

Specimen	IC_50_ (μg/mL)	
	Hypoxanthine as the Substrate	Xanthine as the Substrate
Fraction E1	40.55 ± 1.35 ^a^	51.84 ± 0.75 ^A^
Fraction E2	– *	–
Fraction E3	–	–
Fraction E4	–	–
Fraction E5	–	–
Fraction E6	–	–
Allopurinol **	0.13 ± 0.01 ^b^	0.11 ± 0.01 ^B^

Results are mean ± SD (*n* = 3). * IC_50_ > 80 μg/mL. ** Positive control. Different letters in the table indicate significantly different IC_50_ values between specimens in the same substrate at the level of *p* < 0.05 according to Scheffe’s test.

**Table 3:** Compositions of *Pistacia chinensis* leaf essential oil and fraction E1.

RT (min)	AI	rAI	Constituent	Formula	Content (%)	
					Essential Oil	E1
7.24	932	932	α-Pinene	C_10_H_16_	5.18	2.38
7.90	948	946	Camphene	C_10_H_16_	0.36	-
8.85	972	969	Sabinene	C_10_H_16_	8.25	1.50
9.06	977	974	β-Pinene	C_10_H_16_	1.03	-
9.61	991	990	β-Myrcene	C_10_H_16_	1.23	1.19
10.50	1009	1008	3-Carene	C_10_H_16_	5.32	7.04
10.93	1016	1014	α-Terpinene	C_10_H_16_	4.39	-
11.33	1023	1020	*p*-Cymene	C_10_H_14_	1.32	1.63
11.57	1028	1024	Limonene	C_10_H_16_	24.01	61.06
11.97	1034	1032	*cis*-β-Ocimene	C_10_H_16_	9.15	2.91
12.53	1044	1044	*trans*-β-Ocimene	C_10_H_16_	2.94	0.95
13.14	1055	1054	γ-Terpinene	C_10_H_16_	6.30	1.07
14.66	1081	1086	Terpinolene	C_10_H_16_	1.96	0.53
20.48	1175	1174	Terpinen-4-ol	C_10_H_18_O	9.18	-
21.45	1191	1186	α-Terpineol	C_10_H_18_O	0.71	-
33.86	1380	1389	β-Elemene	C_15_H_24_	1.22	-
35.79	1410	1416	β-Caryophyllene	C_15_H_24_	5.08	3.72
38.34	1447	1452	α-Humulene	C_15_H_24_	0.35	-
Monoterpenes (%)					71.44	80.26
Oxygenated monoterpenes (%)					9.89	-
Sesquiterpenes (%)					6.65	3.72
Total identified (%)					87.98	83.98

RT: Retention time (min); AI: arithmetic index relative to *n*-alkane (C9–C21) on a DB-5MS column; rAI: arithmetic index on a DB-5MS column in the reference [44]; identification methods: MS and AI.

**Table 4:** IC_50_ values of main compounds of fraction E1 against xanthine oxidase.

Specimen	IC_50_	
	Hypoxanthine as the Substrate	Xanthine as the Substrate
Limonene	37.69 ± 2.52 *^,b^ (0.28 ± 0.02) **	48.04 ± 0.78 ^A^ (0.35 ± 0.01)
3-Carene	110.34 ± 1.73 ^a^ (0.81 ± 0.01)	>200
Allopurinol	0.15 ± 0.02 ^c^ (1.10 ± 0.15) ***	0.12 ± 0.02 ^B^ (0.88 ± 0.15) ***

*: μg/mL; **: mM; ***: μM; Allopurinol: positive control; different letters in the table represent significantly different IC_50_ values between specimens in the same substrate at the level of *p* < 0.05 according to Scheffe’s test.

**Table 5:** Kinetic parameters of allopurinol against xanthine oxidase in enzyme kinetic study.

**Substrate**	**Kinetic Parameter**	**Concentration (μg/mL)**					**Potential**	**Inhibition** **Type**
		0.000	0.313	0.625	1.250	2.500		
Hypoxanthine	V_max_	0.00025	0.00025	0.00024	0.00024	0.00024	― *	Competitive
	K_m_	0.00178	0.01655	0.02007	0.02576	0.03277	↑ **	
Xanthine	V_max_	0.00027	0.00026	0.00026	0.00025	0.00026	― *	Competitive
	K_m_	0.00165	0.01369	0.02060	0.02731	0.03867	↑ **	

*: constant; **: increasing.

**Table 6:** Kinetic parameters of limonene against xanthine oxidase in enzyme kinetic study.

Substrate	Kinetic Parameter	Concentration (μg/mL)					Potential	Inhibition Type
		0	40	50	100	200		
Hypoxanthine	V_max_	0.00030	0.00027	0.00025	0.00024	0.00023	↓ *	Mixed
	K_m_	0.00232	0.00407	0.00552	0.00603	0.00670	↑ **	
Xanthine	V_max_	0.00033	0.00029	0.00026	0.00022	0.00020	↓ *	Mixed
	K_m_	0.00181	0.00245	0.00294	0.00359	0.00394	↑ **	

*: decreasing; **: increasing.

## Data Availability

The data are available from the corresponding author on reasonable request.
